# IgG4 Related Lung Disease

**DOI:** 10.1155/2016/1409281

**Published:** 2016-03-30

**Authors:** Mihir Patel, Beena Kumar, My Linh Diep, Deepali Nandurkar

**Affiliations:** Monash Imaging, Monash Health, 246 Clayton Road, Clayton, VIC 3168, Australia

## Abstract

IgG4 related disease is a poorly understood immune mediated condition. Lung involvement is rare and difficult to diagnose and can mimic primary lung malignancy on imaging. A patient who was found to have an incidental lung lesion with risk factors for primary pulmonary malignancy is reported.

## 1. Case Presentation

A 70-year-old woman was found to have an incidental left lower lobe lung mass on chest radiograph in setting of lower respiratory tract infection symptoms. She is an ex-smoker but with long history of smoking since the age of 18 years. Past medical history includes breast cancer that was treated with wide local excision and nodal dissection 7 years earlier. Apart from very short duration of lower respiratory tract symptoms patient denied history of chronic cough or hemoptysis. However, patient revealed 14 kilograms of weight loss over a period of 4 years.

**Figure 1:**
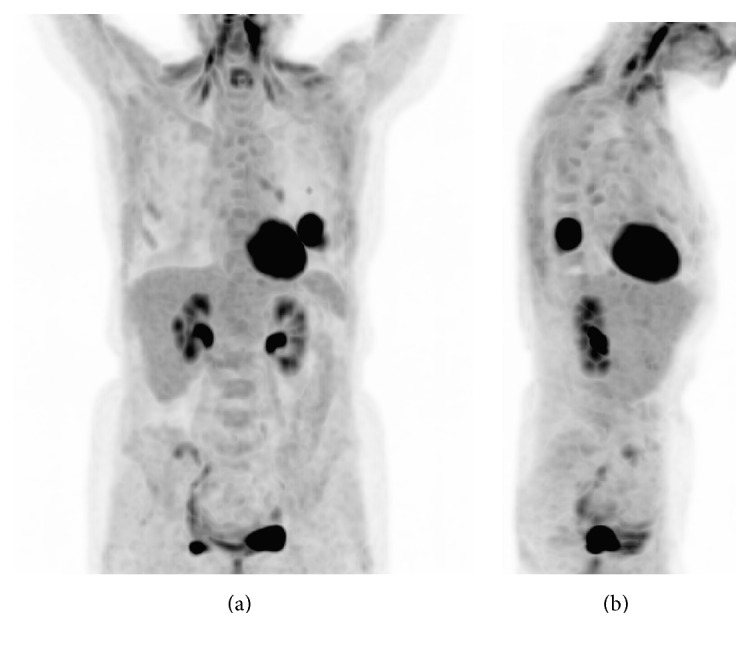
Coronal and lateral maximum intensity projection (MIP) images of the PET showing intensely FDG avid left lung mass and associated left hilar lymphadenopathy.

**Figure 2:**
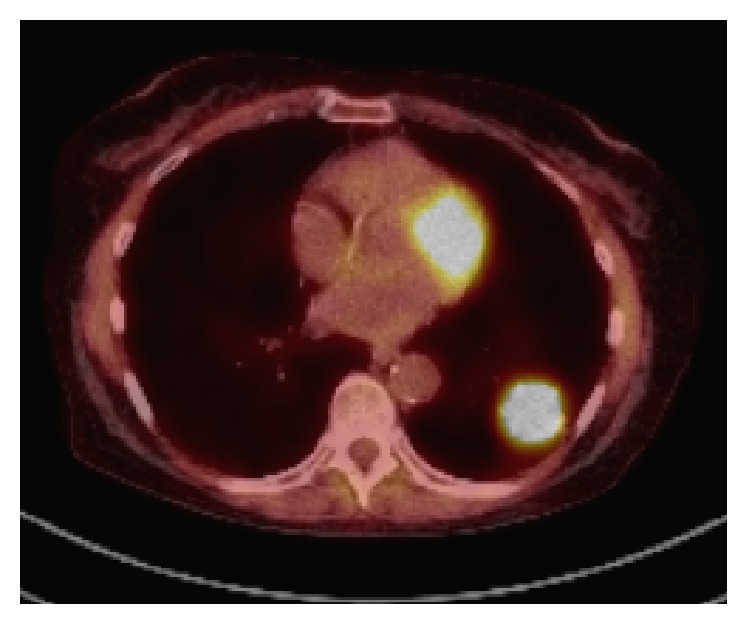
Fused PET/CT axial image shows the left lung mass.

Further investigation with computed tomography (CT) demonstrated a 3.9 cm spiculated mass in the left lower lobe with an 8 mm satellite nodule. Patient subsequently underwent a positron emission tomography (PET) scan that demonstrated an intensely avid left lower lobe mass with standardized uptake value (SUV) max of 18.82. Satellite nodule, ipsilateral hilar, and mediastinal lymph nodes showed low-grade FDG uptake (Figures 1(a), 1(b), and 2). These findings were suggestive of primary lung malignancy with T3, N2, and M0 disease.

Patient's serum IgG count was normal 10 g/L with normal reference range of 7.5−15.6 g/L. Serum IgG4 test was not performed. Patient underwent a CT guided biopsy of the left lower lobe mass that demonstrated inflammatory fibroblastic lesion. However due to high clinical suspicion of primary lung malignancy, left lower lobectomy was performed. Histological examination revealed lymphoplasmacytic infiltration with mainly IgG4 positive plasma cells. Hence IgG4 related pulmonary inflammatory pseudotumour was diagnosed.

## 2. Discussion

IgG4 related disease is a poorly understood immune mediated condition consisting of group of disorders that share specific pathologic, serologic, and clinical features [1]. Common features include tumour-like swelling of involved organs, lymphoplasmacytic infiltrate with IgG4 positive plasma cells, variable amount of fibrosis, and elevated serum level of IgG4 in up to 60% of patients. IgG4 related disease is most frequently seen in middle aged and older men.

Type 1 autoimmune pancreatitis is the most common manifestation of IgG4 related disease. Other common IgG4 related diseases include sclerosing cholangitis, dacryoadenitis/sialadenitis, idiopathic retroperitoneal fibrosis, interstitial pneumonitis, and pulmonary inflammatory pseudotumours to name a few [2].

IgG4 related lung disease has been reported in as many as 13% of patients with autoimmune pancreatitis [3]. Variety of pulmonary involvement has been demonstrated on imaging, including mass-like lesions, bronchovascular nodular pattern which may mimic sarcoidosis, round area of ground glass opacification that may mimic adenocarcinoma in situ, diffuse ground glass opacification that may be mistaken for nonspecific interstitial pneumonia, and air-space consolidation [4, 5].

Parenchymal mass-like lesions or nodules often have spiculated margins and are confined to one lobe, thus mimicking a primary pulmonary malignancy. Enlargement of hilar or mediastinal lymph nodes is also common in patients with IgG4 related lung disease. PET imaging can demonstrate intense FDG uptake in mass-like lesions as well as the nodal disease. Pulmonary disease can be isolated with no involvement of other organs; however, extrapulmonary involvement, most commonly type 1 autoimmune pancreatitis, often precedes pulmonary disease [3].

Pathogenesis of IgG4 related disease is not well understood. Diagnosis of IgG4 related disease requires characteristic findings upon biopsy of affected tissue. Pathological diagnosis criteria include dense lymphoplasmacytic infiltrate, “storiform” fibrosis, and obliterative phlebitis [6]. “Storiform” pattern fibrosis of connective tissue is typified by a cartwheel appearance of arranged fibroblasts interspersed with variable number of inflammatory cells depending in part on the tissue of origin with fewer plasma cells on average in lung pathology.

Compared to other organ systems of pathology, lung IgG4 related disease has fewer plasma cells per high power field, infrequent findings of “storiform” fibrosis and obliterative phlebitis, and a more common finding of obliterative arteritis.

There is a recent international consensus for management and treatment of IgG4 related disease which states that all symptomatic patients and a subset of asymptomatic patients require treatment. Glucocorticoids are the first line agent for active disease, unless there are contraindications for such treatment. Some patients may require additional steroid-sparing immunosuppressive agent, including B-cell depletion agent such as Rituximab, from the start of treatment. Patients with organ threatening IgG4 related disease and with an increased risk of relapse (e.g., multiorgan involvement) are likely to benefit from maintenance therapy after a successful induction therapy. For patients who relapse after cessation of successful initial therapy with clinical remission, retreatment with glucocorticoids is indicated. Steroid-sparing agent for continuation in the remission maintenance period should also be considered [7].

Natural history of IgG4 related disease is not clear. Most patients respond to treatment. However, relapses are common. Further organs and tissues may become involved, sometimes despite apparently effective treatment. Although no definite causal relationship has been established, several types of lymphomas have been reported in patients with IgG4 related disease [8].

Our case demonstrates IgG4 related lung disease mimicking high-grade primary lung malignancy on structural and functional imaging. It is a rare condition that can be mistaken for lung malignancy on imaging particularly when there is isolated pulmonary involvement. IgG4 related disease could involve multiple organ systems. Pulmonary involvement is usually preceded by another organ involvement most commonly autoimmune pancreatitis.

## Learning Objectives

Recognize that although IgG4 related lung disease is rare, it can mimic primary lung malignancy on structural and functional imaging.

Appreciate IgG4 related disease can often involve multiple organs.

Pretest includes the following questions:

 What is the pathophysiology of IgG4 related disease?
 Which extrapulmonary organs may be involved in IgG4 related disease?

Posttest includes the following questions: 

*What Is the Pathophysiology of IgG4 Related Disease*? IgG4 related disease is a poorly understood immune mediated condition consisting of group of disorders that share specific pathologic, serologic, and clinical features. Common features include tumour-like swelling of involved organs, lymphoplasmacytic infiltrate with IgG4 positive plasma cells, variable amount of fibrosis, and elevated serum level of IgG4 in up to 60% of patients. Pathological diagnosis criteria include dense lymphoplasmacytic infiltrate, “storiform” fibrosis, and obliterative phlebitis. 

*Which Extrapulmonary Organs Are Frequently Involved in IgG4 Related Disease*? Pancreas is the most common organ involved in IgG4 related disease with autoimmune pancreatitis. Other IgG4 related diseases include biliary disease with sclerosing cholangitis, retroperitoneal involvement including retroperitoneal fibrosis, and lacrimal/salivary gland inflammation.
